# Progressive non-infectious anterior vertebral fusion, split cord malformation and situs inversus visceralis

**DOI:** 10.1186/1471-2474-7-94

**Published:** 2006-12-05

**Authors:** Ali Al Kaissi, Farid Ben Chehida, Maher Ben Ghachem, Franz Grill, Klaus Klaushofer

**Affiliations:** 1 Ludwig Boltzmann Institute of Osteology at the Hanusch Hospital of WGKK and AUVA Trauma Centre Meidling, 4th Medical Department, Hanusch Hospital. Heinrich Collins Str. 30 A-1140, Vienna, Austria; 2 Department of Paediatric Orthopaedic Surgery-Children Hospital of Tunis, Jabari, 1007, Tunisia; 3 Center of Radiology-Department of Imaging Studies-Ibn Zohr Institute, Tunis, City Khadra 1003, Tunisia; 4 Orthopaedic Hospital of Speising, Paediatric Department, Speisinger Str. 109, Vienna-1130, Austria

## Abstract

**Background:**

Progressive non-infectious anterior vertebral fusion is a unique spinal disorder with distinctive radiological features. Early radiographic findings consist of narrowing of the anterior aspect of the intervertebral disk with adjacent end plate erosions. There is a specific pattern of progression. The management needs a multi-disciplinary approach with major input from the orthopaedic surgeon.

**Case report:**

We report a 12-year-old-female with progressive anterior vertebral fusion. This occurred at three vertebral levels. In the cervical spine there was progressive fusion of the lateral masses of the Axis with C3. Secondly, at the cervico-thoracic level, a severe, progressive, anterior thoracic vertebral fusion (C7-T5) and (T6-T7) resulted in the development of a thick anterior bony ridge and massive sclerosis and thirdly; progressive anterior fusion at L5-S1. Whereas at the level of the upper lumbar spines (L1) a split cord malformation was encountered. Situs inversus visceralis was an additional malformation. The role of the CT scan in detecting the details of the vertebral malformations was important. To our knowledge, neither this malformation complex and nor the role of the CT scan in evaluating these patients, have previously been described.

**Conclusion:**

The constellations of the skeletal abnormalities in our patient do not resemble any previously reported conditions with progressive anterior vertebral fusion. We also emphasise the important role of computerized tomography in the investigation of these patients in order to improve our understanding of the underlying pathology, and to comprehend the various stages of the progressive fusion process. 3D-CT scan was performed to improve assessment of the spinal changes and to further evaluate the catastrophic complications if fracture of the ankylosed vertebrae does occur. We believe that prompt management cannot be accomplished, unless the nature of these bony malformations is clarified.

## Background

Progressive non-infectious anterior vertebral fusion is a unique spinal disorder with distinctive radiological features. Early radiographic findings consist of narrowing of the anterior aspect of the intervertebral disk with adjacent end plate erosions. There is a specific pattern of progression. The management needs a multi-disciplinary approach with major input from the orthopaedic surgeon.

## Case presentation

**Figure 1:**
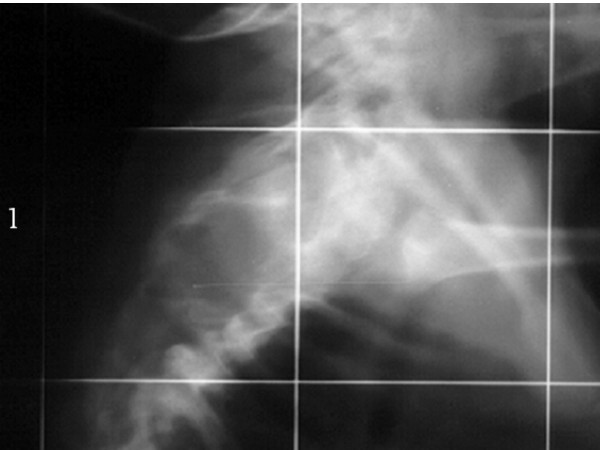
Lateral radiogram showed the severe kyphosis.

The child was referred to our department at the age of 12 years because of progressive thoraco-lumbar kyphoscoliosis and progressive limitations of neck movement (fig 1). She was born at full term, the product of an uneventful gestation. At birth her length, weight, and OFC were around the 10^th ^percentile. The mother was a 27-year-old-healthy woman, gravida 1 abortus 0, married to a 32-year-old unrelated man.

At birth the parents observed a patch of hair over the lumbar region, and the child was investigated for this. A split cord malformation was identified, but the parents refused further interventions. At the age of 9 years the parents observed marked worsening of the spinal tilting and problems in bending over. Her head movements became difficult, particularly flexion, and this was accompanied by pain, more marked in the occipital and suboccipital regions. Walking a distance was difficult.

**Figure 2:**
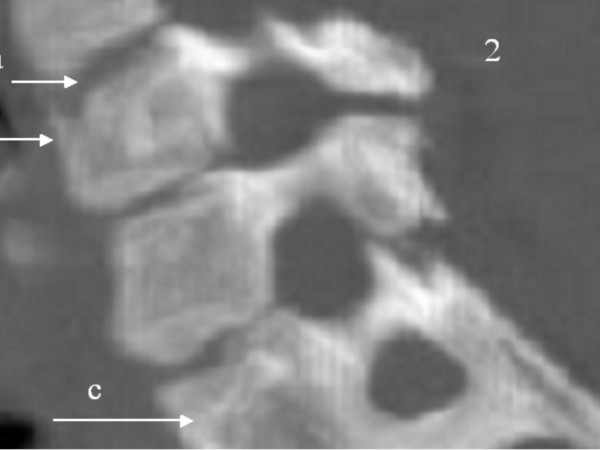
Early stage of progressive vertebral fusion in which C4-C6, showed progressive anterior disc narrowing and end plate irregularities (arrows; a-b), whereas (arrow c) showed the development of a thick anterior and posterior bony ridge.

**Figure 3:**
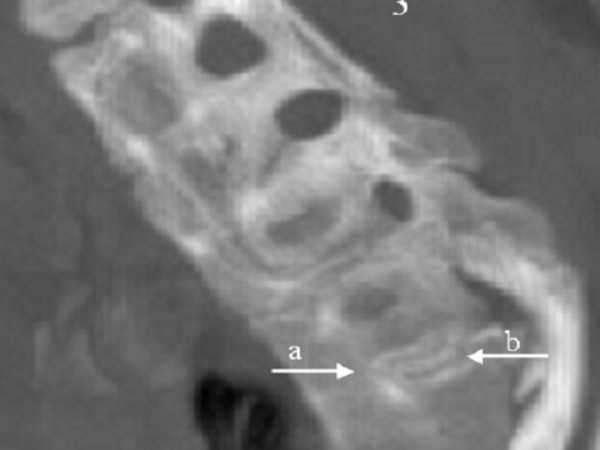
End stage of the progressive anterior vertebral fusion and the multi-level anterior fusion with disc space obliteration (T1-T5). There is a massive bony ridge extending anteriorly and in some vertebrae, posteriorly as well. However, (arrow b) note the sparing of the disc space posteriorly, whereas the anterior end plate is totally obliterated (arrow a). Absence of the normal concavity of the anterior body surface. There is a proliferation of sclerotic bone.

**Figure 4:**
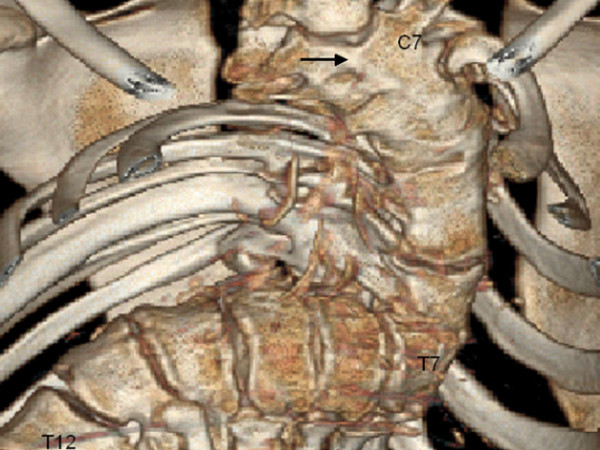
3 d reconstruction CT scan showed the massive anterior fusion of (C7-T5) and (T6-T7), and the apparent anterior thick bony ridge (arrow), the latter developed secondary to progressive ossification of the anterior longitudinal ligament. from T7-T12; note the narrowing of the anterior part of the disc space, accompanied by erosion and irregularity of the anterior end plates.

**Figure 5:**
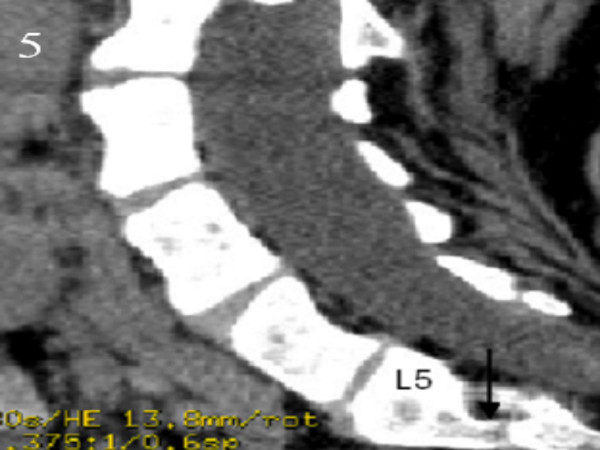
Note sparing of the lumbar spines and progressive anterior fusion of L5-S1, and the exaggerated lumbar lordosis secondary to massive fusions of the thoracic vertebrae.

**Figure 6:**
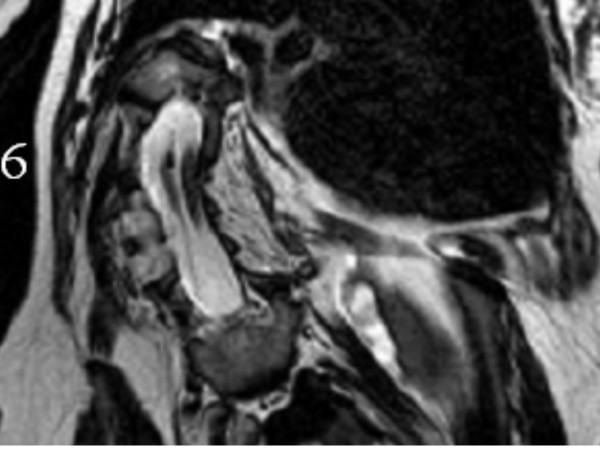
sagittal MRI imaging showed a split cord malformation, atthe level of L1, with a bony bar at the L1 level. It also revealed situs inversus visceralis. MRI imaging of the brain did not show any abnormality, and sagittal MRI of the cervical region did not reveal any Arnold-Chiari malformation. Other imaging studies such, as echo-cardio-Doppler was normal. The pelvic ultrasound showed normal ovaries, uterus and vagina, and renal ultrasound showed a normal genito-urinary system.

Her subsequent course of development has been described as within the normal limits, except for a moderate delay in motor development. There was no history of serious illnesses. Physical examination at the age of 12 years revealed; short stature. Her height was 121 cm (-3SD) and her head circumference was 53 cm (+2SD). She was of normal intelligence, and neurological examination, apart from a neuropathic bladder was unremarkable. Hearing and vision were normal. Stiffness of the neck was noted, with limitation of neck movement, particularly in flexion. Musculo-skeletal examination showed relative ligamentous hyperlaxity in the limbs, normal hands and feet, and the skeletal survey did not reveal limb abnormalities. The spinal column showed; severe, rigid, thoraco-lumbar kyphoscoliosis (fig 2, 3, 4, 5, 6).

Laboratory tests included hematological indices, urine screening for metabolic abnormalities, karyotype (for the child and her parents) and rhematological screening. These were all normal and the HLA B-27 was negative.

Family history was unremarkable. Parents were reluctant to give any relevant information.

Progressive, non-infectious anterior vertebral fusion is a rare disorder; which is often referred to as the Copenhagen syndrome [1]. In the classical form, there may be a characteristic anterior defect in the affected vertebrae from shortly after birth, associated with narrowing of the anterior part of the disc space. This is accompanied by erosion and irregularity of the anterior end plates, and when the process of narrowing progresses with age, there is eventual disc space obliteration and bony ankylosis anteriorly, via a thick bony ridge [2].

It is important, to differentiate progressive non-infectious anterior vertebral fusion from the congenital form of block vertebrae, firstly by its clinical history and secondly, by using CT scanning (fig 3, 4, 5)

Scoliosis and kyphoscoliosis in children can occur either in isolation or as a part of a number of syndromes. Spinal malsegmenatation occurs in many of these syndromes, and this includes spondylocarpotarsal synostosis, spondylothoracic dysplasia, and other rare conditions [3,4]. All of these disorders have characteristic patterns of vertebral malformation, such as a posterior unsegmented spinal bar, congenital block vertebra, carpal and tarsal malformations [5].

Knutsson et al., [6] and others [1,2,7] reported children with progressive anterior vertebral fusion as the only malformation, and in none was this part of a syndrome.

Hughes et al., [8] reported three children with progressive fusion, and other congenital and developmental abnormalities. However, there were no distinctive clinical or radiological features signifying a syndromic association, apart from one child, who presented with spinal dysraphism, but neither cervical vertebral fusion nor situs inversus visceralis were described. Philip et al. [9] described involvement of both the upper thoracic (T2-T5) and lower thoracic (T10-S1) vertebrae, associated with radio-ulnar synostosis, exostosis, short and broad clavicles, and a balanced t(10;20)(p11;p13) translocation. There was no cervical vertebral fusion, spilt cord malformation or situs inversus visceralis.

Farrior et al., [10] described a male child with progressive vertebral fusion of the cervical, thoracic, and lumbar vertebrae, with additional manifestations, such as absence of one cervical vertebra, clefting of the vertebral bodies, and other few minor non-spinous abnormalities. The overall features were different from these found in our patient.

Fryns et al., [11], described a child who presented with progressive anterior vertebral body fusion, and other abnormalities such as a generalised overgrowth, especially of the hands and feet, and unusually thick skin and subcutaneous tissue of upper and lower limbs. There was facial dysmorphism. These features were not seen in our patient.

Tubbs et al., [12,13] reported split cord malformation in association with Klippel-Feil syndrome, and another child presented with split cord malformation and situs inversus totalis and scoliosis. They focused on the possibility that defects of the midline and laterality defects (situs inversus) are etiologically related. However, the reported patient manifested congenital blocked vertebrae and not the progressive condition as described here (fig 3, 4, 5, 6).

McRae and Barnum [14] reviewed 25 patients who presented with atlanto-occipital fusion. They found a bony continuity between the anterior arch of the atlas and the anterior lip of the foramen magnum. However, it is uncertain given the absence of sophisticated imaging techniques whether the bony continuity was because of progressive fusion, or was congenital. Situs inversus was not documented.

Kalifa et al., [15] described the nature of progressive non-infectious vertebral fusion, in which the lesions involved mainly the anterior end plates and sparing the posterior parts, whereas in congenital vertebral blocking (failure of segmentation) it usually involves the posterior part of the disk.

## Conclusion

The constellations of the skeletal abnormalities in our patient do not resemble any previously reported conditions with progressive anterior vertebral fusion. We also emphasise the important role of computerized tomography in the investigation of these patients in order to improve our understanding of the underlying pathology, and to comprehend the various stages of the progressive fusion process. 3D-CT scan was performed to improve assessment of the spinal changes and to further evaluate the catastrophic complications if fracture of the ankylosed vertebrae does occur. We believe that prompt management cannot be accomplished, unless the nature of these bony malformations is clarified.

## Competing interests

The author(s) declare that they have no competing interests.

## Authors' contributions

All authors contributed to this work and all read and approved the final manuscript.

A A: Was responsible for, writing the manuscript, Conception and design and data analysis.

F B C, and M B G: Data analysis.

F G and K K: Conception and design.

## Pre-publication history

The pre-publication history for this paper can be accessed here:
